# Burnout trajectories among healthcare workers during a pandemic, and predictors of change

**DOI:** 10.1186/s12913-025-12802-w

**Published:** 2025-05-27

**Authors:** Kristina Bondjers, Philip Hyland, Dan Atar, Jan Olav Christensen, Kristian Bernhard Nilsen, Solveig Klaebo Reitan, Leiv Arne Rosseland, Tore Wentzel-Larsen, Hilde Wøien, John Anker Zwart, Grete Dyb, Synne Stensland

**Affiliations:** 1 Norwegian Centre for Violence and Traumatic Stress Studies, Oslo, Norway; 2 Department of Psychology, Maynooth University, Kildare, Ireland; 3 Institute of Clinical Medicine, Faculty of Medicine, University of Oslo, Oslo, Norway; 4 Department of Research and Innovation, Division of Clinical Neuroscience, Oslo University Hospital, Oslo, Norway; 5 Section for Clinical Neurophysiology, Department of Neurology, Oslo University Hospital, Oslo, Norway; 6 Department of Mental Health, Faculty of Medicine and Health Sciences, NTNU, Trondheim, Norway; 7 Nidelv District Psychiatric Center, St Olavs Hospital, Trondheim, Norway; 8 Department of Cardiology, Division of Medicine, Oslo University Hospital Ullevål, Oslo, Norway; 9 Division of Emergencies and Critical Care, Oslo University Hospital, Oslo, Norway; 10 Department of Research and Development, Division of Emergencies and Critical Care, Oslo University Hospital, Oslo, Norway; 11 Group for Work Psychology and Physiology, National Institute of Occupational Health, Oslo, Norway; 12 Department of Public Health Science, Faculty of Medicine, University of Oslo, Oslo, Norway

**Keywords:** Latent growth curve model, Burnout, Covid-19 pandemic, Healthcare workers

## Abstract

**Background:**

While several cross-sectional studies have suggested high burnout levels among health-care workers (HCW) during the Covid-19 pandemic, fewer studies have examined longitudinal trajectories of burnout.

**Objectives:**

To examine (1) trajectories of change in levels of burnout among Norwegian HCW during a one-year period in the mid-phase of the Covid-19 pandemic (second, third, and fourth incidence rate peaks), and (2) how demographic and occupational variables, and pandemic-related experiences (e.g., own infection, severe infection in family, friend, or colleague, caring for deceased patient with Covid-19) were associated with such change.

**Methods:**

Burnout was measured by the Copenhagen Burnout Inventory. Latent growth curve modeling was used to estimate trajectories of burnout symptoms, and predictors of starting point and rate of change in burnout levels.

**Results:**

Starting points of burnout scores were, on average, low-to-moderate. Women, younger HCW, those living alone, and nurses had higher initial scores. Overall, burnout scores remained mostly unchanged across the study period. However, lower burnout scores at the initial assessment were associated with increasing scores over time. Being exposed to patients with Covid-19 and having a Covid-19 infection were associated with increased burnout over time.

**Conclusions:**

While burnout symptoms among frontline health workers remained stable across the peaks of the Covid-19 pandemic overall, the study identified higher risk of worsening symptoms over time among certain demographic (younger personnel, females, and nurses) and highly exposed individuals and groups. These findings may be helpful for identifying frontline workers at particular risk of burnout during future public health emergencies.

## Introduction

Identifying risk factors for burnout among frontline health care workers (HCWs) during prolonged public health emergencies is essential to strengthen preparedness planning and response. The occupational phenomenon of burnout is included in the 11th version of International Classification of Diseases (ICD-11) in the chapter “Factors influencing health status or contact with health services” [1] Burnout refers to an individual’s emotional and physical response (fatigue, energy depletion, emotional exhaustion) to chronic occupational stress [2, 3]. During public health emergencies, such as the Covid-19 pandemic, the increased and prolonged stress faced by HCWs could potentially enhance the risk of developing burnout. Indeed, a recent systematic review looking at cross-sectional studies published up until February 28, 2021, found that up to 90% of HCWs were suffering from burnout, although considerable variation existed across studies [4].

Burnout is seen as a continuing and dynamic process developing in response to environmental stressors, and should theoretically unfold over time [5]. Despite this, both before and during the Covid-19 pandemic, longitudinal studies on burnout among HCWs have been sparse, and results mixed [4, 6, 7]. In relation to the pandemic, several repeated cross-sectional and some longitudinal studies indicated that prevalence and symptom levels of burnout increased while others found diminished rates or no change [8–11]. For example, in a sample of Dutch HCWs tracked from before to during the outbreak of Covid-19 (up until November 2021), symptoms of emotional exhaustion remained nearly unchanged despite worsening work conditions [12]. In a nine-country study examining change in burnout from mid-2020 to early 2021, levels of burnout increased, particularly for HCWs in close contact with patients with Covid-19 [13]. During the first year of the pandemic, a one-site study in New York suggested that early and persistent burnout was strongly predicted by pre-pandemic burnout levels, whereas burnout onset at a later time-point was associated with, among other predictors, caring for patients that deceased with Covid-19 [14]. Some longitudinal and repeated cross-sectional studies have suggested that burnout levels appear to increase and coincide with Covid-19 incidence rates [15, 16].

These studies have to some degree examined alterations in levels of burnout across the pandemic. However, most of them used small samples, and few examined intraindividual trajectories of change and between-person differences in such trajectories. Burnout develops over time and should theoretically increase with prolonged stress. Thus, knowledge of the predictors of development, trajectories, and recovery of burnout among HCWs could help guide healthcare organizations in their preparedness planning and development of targeted interventions to meet the needs of frontline personnel responding to future public health emergencies. Thus, in the current study, we aimed to examine (1) change in levels of burnout among a large sample of HCWs during a one-year period in the middle phase of the pandemic, and (2) how demographic (i.e., gender, age, living alone), occupation (i.e., profession, geographical work area, working with patients with Covid-19) and adverse pandemic-related experiences (e.g., own infection, severe infection in family, friend, or colleague, caring for deceased patient with Covid-19) are associated with change in symptoms of burnout.

## Methods

### Participants and procedure

This study draws upon data collected during a longitudinal open-cohort study investigating pandemic exposure, work environment and mental health across the Covid-19 pandemic in Norway. Participants were HCWs at four large university hospitals (Oslo University Hospital (OUS), Akershus University Hospital (AHUS), St Olavs Hospital (St Olav), and the University Hospital of North Norway (UNN)) in different geographical regions of Norway. All participating hospitals were responsible for care of patients with Covid-19 in their respective regions, and thus, all participants are considered frontline workers. Eligible participants were recruited to partake in a web-based survey using the standard mode for communication (e.g., email, SMS, online bulletin boards) used by the participating hospitals. Data collections were performed in conjunction with incidence rate peaks in Norway. The present analysis focuses on HCWs who participated in the study at the second peak: December 2020 (*N* = 1088, 75% women, Mean age = 42 (SD = 11). Of these, 546 had at least one follow-up assessment; 317 participated in a survey in April 2021 and 293 in January 2022; and 153 participants participated at all three time-points (information on missing data analysis is presented in the Results section). Almost half (45%) worked as nurses, 18% as physicians, and the remaining were in other positions at the hospitals (e.g., biotechnicians, physiotherapists, psychologists, nutritionists, ambulance personnel).

### Measures

#### Predictors

##### Participants’ demographic and professional characteristics

Participants’ age at the second wave and sex were derived from their social security number. Their profession was categorized as ‘nurse’, ‘physician’ or ‘other frontline worker’, with the latter including all other hospital personnel. Participants were categorized as living alone (with or without children < 18 years old) or living with another adult, based on their own report. Primary workplace was dichotomized based on geographic differences in incidence rates into South-east Norway (AHUS and OUS) and Mid-north Norway (UNN and St Olav). Overall, across the pandemic, cumulative rates of Covid-19 were lower in Mid-north Norway compared to South-east Norway [17].

##### Level of contact with patients with Covid-19

Exposure to patients with Covid-19 was determined based on participants’ self-reported interactions with individuals suspected or diagnosed with Covid-19 during the pandemic. Participants were categorized as either “Direct contact” (i.e., having been in physical contact with patients with suspected or diagnosed disease) or “Indirect contact” (i.e., working at the hospitals during the outbreak, but reporting no direct contact with suspected or diagnosed Covid-19 patients). This classification aligns with recommendations provided by Pollock et al. [18].

##### Potentially traumatic pandemic events

Participants reported on whether they had (1) been responsible for the treatment of a severely ill patient(s) with Covid-19 (2), been responsible for treatment of (a) patient(s) that deceased with Covid-19 (3), experienced death of a friend, family member, or colleague due to Covid-19 (4), being infected by Covid-19, and (5) having a friend, family or colleague with severe Covid-19 infection requiring hospitalization. during the study period (all coded yes or no).

#### Outcome

##### Burnout

Burnout was assessed using four items derived from the personal burnout subscale in the Copenhagen Burnout Inventory (CBI) [19]. CBI has been proposed as a gold-standard measure for the assessment of burnout among healthcare, across multiple reviews of psychometric measures [20, 21]. The CBI personal burnout subscale assesses the degree of physical and psychological fatigue and exhaustion experienced by the respondent. The subscale originally consisted of six items. However, due to the nature of the stressor at hand (i.e. the pandemic) and to avoid overburdening the participants, only four of these items were included. The excluded items were “How often do you feel tired” and “How often do you feel weak and susceptible to illness” and were removed based on the belief that they would reflect fear of, or actual infection. Respondents were asked how often they experienced a symptom on a 5-point Likert scale (0 = never, 100 = (almost) every day). As the developers of the original 6-item instrument suggest that a mean score may be calculated as long as respondents have answered at least three of the six items we decided to calculate scores based on the mean of participants’ responses for participants responding to at least three of the four included items (range 0–100). Average score for personal burnout in the original evaluation of the CBI was 32.7 (SD = 15.7). In a longitudinal study of Norwegian ICU personnel average scores for personal burnout was 16.7–20.8 [22], and among Norwegian midwives 34.26 [23]. A sum score ≥ 50 is considered indicative of burnout syndrome [19, 24]. The CBI has demonstrated good psychometric properties in terms of reliability, and concurrent and predictive validity [19]. In the current sample, the internal reliability of the CBI scores was good (α = 0.85).

### Statistical analysis

An ‘unconditional’ latent growth curve model (i.e., one with no predictor variables) was first estimated to determine if change in burnout scores across the one-year study period followed a linear trajectory. As data were only available at three time periods, it was not possible to test alternative descriptions (i.e., a quadratic model) of change in burnout scores. If the unconditional linear growth curve model fit the sample data, the model would be extended to include the demographic and occupation variables as predictors of the intercept (i.e., the starting point) and the adverse pandemic related experiences were added as predictors of the slope (i.e., rate of change) latent variables of burnout. These analyses were conducted in Mplus version 8.1 [25].

Model fit was judged according to standard guidelines [26] where good model fit is indicated by a non-significant chi-square (*χ*^*2*^) result, Comparative Fit Index (CFI) and Tucker-Lewis Index (TLI) above 0.90 and closer to 1, and Root Mean Square Error of Approximation (RMSEA) and Standardized Root Mean Square Residual (SRMR) values below 0.08 and closer to 0. These models were estimated using robust maximum likelihood estimation (MLR), and missing data were handled via full information maximum likelihood estimation.

Time-independent predictors of the intercept and slope latent variables were sex (0 = females, 1 = males), age, living alone (0 = no, 1 = yes), hospital region in Norway (0 = Mid-north, 1 = South-east), and type of healthcare professional (dummy coded variables representing being a nurse and a physician versus ‘other frontline HCW’). Several other time-dependent variables were used to predict the slope latent variable only (n.b., these variables were not used to predict the intercept variable because they were measured across the study period and therefore could not predict initial burnout scores). These predictor variables were being directly exposed to patients with Covid-19 during the study period (0 = no, 1 = yes), treated a severely ill patient(s) with Covid-19 during the study period (0 = no, 1 = yes), treated a patient(s) that died from Covid-19 during the study period (0 = no, 1 = yes), experienced the death of a friend, family member, or colleague due to Covid-19 during the study period (0 = no, 1 = yes), becoming infected by Covid-19 during the study period (0 = no, 1 = yes), and had a friend, family or colleague experience a severe Covid-19 infection during the study period (0 = no, 1 = yes).

## Results

### Missing data

We initially examined patterns of missing data (Table 1). There was no significant difference in initial burnout scores between those who responded at all three waves and those who did not (‘non-completers’ M (SD) = 27.3 (23.9), ‘completers’ M (SD) = 29.7 (25.9), d = −0.10, *p* = .270). Additionally, non-completers and completers did not differ significantly in their profession, nor - at T2 - in the amount who reported working with patients with Covid-19, experiencing death of a colleague, a close friend or family, severe infection in a colleague, a close friend, or family, or own infection with Covid-19.

**Table 1:** Comparison of non-completers and completers

Baseline variable		Non-completers (< 3 timepoints)	Completers (all 3 timepoints)	Effect size		
		M (SD)	M (SD)			
CBI		27.30 (23.80)	29.60 (25.80)		d = − 0.097,	*p* = .270
Age		41.98 (11.58)	45.20 (11.16)		d = − 0.280,	*p* = .001
Gender		Count(expected)	Count (expected)	X^2^(1) = 0.762	Φ 0.026,	*p* = .383
	Women	709 (705)	111 (115)			
	Men	226 (230)	42 (38)			
Profession				X^2^(1) = 1.547	Φ 0.038,	*p* = .462
	Physician	164 (165)	28 (27)			
	Nurse	411 (417)	74 (68)			
	Other	360 (353)	51 (58)			
Geographical region				X^2^(1) = 20.205	Φ = 0.136,	*p* < .001
	Mid-North	359 (334)	30 (54)			
	South-east	576 (600)	123 (98)			
Working directly with patients with Covid-19				X^2^(1) = 7.780	Φ = 0.0.85,	*p* = .005
	No	556 (540)	73 (89)			
	Yes	375 (391)	80 (64)			
Responsible for treatment of severely ill patient(s) with Covid-19				X^2^(1) = 15.556	Φ = 0.120,	*p* < .001
	No	657 (636)	84 (105)			
	Yes	270 (291)	69 (48)			
Responsible for treating patient(s) that deceased with Covid-19				X^2^(1) = 14.850	Φ =. 117,	*p* < .001
	No	814 (799)	116 (131)			
	Yes	116 (131)	37 (22)			
Experiencing death of family, friend or colleague due to Covid-19				X^2^(1) = 2.148	Φ = − 0.045,	*p* = .143
	No	918 (920)	152 (150)			
	Yes	13 (11)	0 (2)			
Covid-19 infection - self				X^2^(1) = 0.27	Φ = − 0.005,	*p* = .870
	No	902 (902)	148 (148)			
	Yes	33 (33)	5 (5)			
Covid-19 infection– family, friend or colleague				X^2^(1) = 1.044	Φ = − 0.031,	*p* = .307
	No	822 (818)	130 (134)			
	Yes	113 (117)	23 (19)			

*CBI* Copenhagen Burnout Inventory, *X*^*2*^ chi-square, *d* cohen’s d, *Φ* phi-coefficient

Completers and non-completers did significantly differ on the following variables: age (non-completers M (SD) = 41.9 (11.6), completers M (SD) = 45.2 (11.1), d = −0.28, *p* = .001); geographical region, with a higher amount of participants from the South-east region being completers (effect size, as assessed with phi-coefficient, Φ = 0.14, *p* < .001); responsibility for treatment of severely ill patients with Covid-19, with a higher amount of those experiencing this being completers (Φ = 0.12, *p* < .001); and responsibility for treatment of deceased patients, with a lower amount of those experiencing this being completers (Φ = 0.12, *p* < .001). As all effect sizes were small, we assumed that the data were missing at random which is a necessary condition for missing data management via full information maximum likelihood estimation. Nonetheless, readers should keep the differences between completers and non-completers in mind when interpreting results.

The unconditional linear growth curve model provided a reasonably close fit to the sample data (*χ*^*2*^ (1) = 7.99, *p* < .001; CFI = 0.978, TLI = 0.935; RMSEA = 0.081; SRMR = 0.027). The mean of the intercept latent variable was 27.52 (95% CI = 26.07 to 29.91, *p* < .001, SD = 24.19) and the variance was 494.25 (CI = 398.90 to 589.56, SD = 1592.90). Thus, the starting point of burnout scores was within the low-to-moderate range (slightly lower than the average score (32.7) in the original evaluation of CBI), but there was substantial variability in these starting points. The estimated slope was 0.44 (CI = −0.48 to 1.36, *p* = .367), and the variance of the slope was 28.51 (CI = −20.55 to 77.57, *p* = .148). Thus, there was a very small increase in burnout scores across the three waves, but this increase was not significant. Furthermore, there was no evidence of variability between individuals in change in burnout scores over time. The correlation between the intercept and slope variables was negative and statistically significant (*r* = − .29, CI = − 0.55 to − 0.01, *p* = .038), meaning that lower burnout scores at the initial assessment were associated with increasing scores over time (and vice-versa).

The model was then extended to include the predictors of the intercept and slope variables, and this model was a close fit to the sample data: *χ*^*2*^ (19) = 24.76, *p* = .169; CFI = 0.992, TLI = 0.983; RMSEA = 0.017; SRMR = 0.035. The standardized effects for each predictor variable on the intercept and slope latent variables are presented in Table 2. Lower initial burnout scores were significantly associated with being male (β = − 0.13, *p* < .001) and older age (β = − 0.20, *p* < .001), whereas living alone (β = 0.09, *p* = .008) and being a nurse (β = 0.08, *p* = .021) were associated with higher initial scores. Increasing burnout scores over time were associated with being exposed to patients with Covid-19 during the study period (β = 0.31, *p* = .047), and having a Covid-19 infection during the study period (β = 0.17, *p* = .043).

**Table 2:** Standardized regression coefficients for the intercept and slope variables of burnout (*N* = 1,063)

	Intercept β [95% CI], (*p*)	Slope β [95% CI], (*p*)
Sex (0 = females, 1 = males)	**− 0.13 [−0.19;−0.06]**,** (< 0.001)**	0.17 [−0.06;0.39], (0.116)
Age	**− 0.20 [−0.26;−0.14]**,** (< 0.001)**	0.00 [−0.18;0.19], (0.970)
Living alone	.**09 [0.02; 0.15]**,** (0.008)**	0.06 [−0.13;0.26], (0.491)
Hospital region (0 = Mid-north, 1 = South-east)	0.01 [−0.06;0.07], (0.855)	− 0.22 [−0.49;0.04], (0.075)
Nurse^1^	**0.08 [0.01;0.16]**,** (0.021)**	− 0.17 [−0.42;0.09], (0.165)
Physician^1^	− 0.04 [−0.10;0.03], (0.283)	− 0.13 [−0.35;0.09], (0.249)
Working directly with patient(s) with Covid-19	--	**0.31 [−0.01;0.64]**,** (0.047)**
Responsible for treatment of severely ill patient(s) with Covid-19	--	− 0.06 [−0.32;0.20], (0.653)
Responsible for treating deceased patient(s) with Covid-19	--	0.04 [−0.15;0.24], (0.658)
Experiencing death of family, friend or colleague due to Covid-19	--	− 0.01[−0.19;0.16], (0.847)
Covid-19 infection - self	--	**0.17 [−0.02;0.37]**,** (0.043)**
Covid-19 infection – family, friend or colleague	--	− 0.06 [−0.21;0.09], (0.464)

^1^ = reference group is ‘any other healthcare worker’; β = standardized beta value; p = statistical significance value; statistically significant effects are in bold

## Discussion

The current study aimed to examine how levels of burnout among HCWs changed over a one-year period starting with the second incidence rate peak of the pandemic in Norway. By design, assessments coincided with high incidence rates of Covid-19. Four key findings emerged. First, levels of burnout early in the pandemic were, on average, relatively low among the HCWs although there was considerable variability between individuals. Second, levels of burnout remained largely unchanged across the 12-month study period (coinciding with the most extreme pandemic conditions), and there was no evidence (*p* = .148) of inter-individual differences in change in burnout scores. There was however a negative association between one’s initial level of burnout and one’s change over time, with HCWs having lower levels of burnout at the initial assessment experiencing an increase over time, and HCWs with higher levels of burnout at the initial assessment experiencing a decrease over time. Third, women, younger HCWs, those living alone, and nurses had higher burnout scores at the initial assessment. Fourth, increased burnout over time was associated with working with patients with Covid-19 and being infected with Covid-19.

The present results that levels of burnout remained stable over time during the pandemic are generally consistent with much of the existing evidence with HCWs. Results from a meta-analysis of cross-sectional studies examining burnout levels among physicians found that burnout was highest during the earliest phase of the pandemic [27]. Theoretically, burnout levels might be expected to increase over time due to chronic exposure to a highly stressful situation but this is not the picture that is emerging in the empirical literature. There are possible explanations for why burnout appears to have remained relatively stable across time among HCWs. Individual resilience strategies and organizational interventions might have mitigated the long-term impact of working under stress. Furthermore, time may have allowed HCWs to learn more about how to treat and manage patients with Covid-19, possibly easing stress levels via settling into routines and learning new skills. In pre-pandemic studies, changes in one’s job (both shifting organizations as well as intra-organizational shifts in work tasks) have been shown to be associated with initial increases in burnout that subsequently stabilizes suggesting that changes in burnout are not only associated with increased demands/pressure, but perhaps also with insecurity and novelty of tasks [5]. Additionally, it is possible that as time progressed and a clearer picture emerged of the lethality of the Covid-19 virus, along with the emergence of Covid-19 vaccines, stress associated with working in this public health context diminished.

On the other hand, the follow-up period of this study (1 year) in the context of unfolding burnout symptoms was fairly short, and it is possible that symptoms may not properly manifest until after cessation of acute exposure. We note however that our findings align with what has been observed regarding changes in anxiety and depression in the general populations across Europe, with overall stable or decreasing levels of anxiety and depression [28].

The finding that initially low levels of burnout were associated with increasing levels over time (vice-versa) could have several explanations. It is possible that individuals with initially higher symptoms were less likely to work in highly stressful circumstances during the subsequent study period, and vice versa, thus leading to a negative association between starting point and change over time. It is also possible that the seemingly reduced inter-individual variability is simply due to regression to the mean.

We found that younger HCWs, women, nurses and people living alone started out with higher levels of burnout compared to other HCWs. Neither age nor sex were significantly related to the course of symptoms over time. Similar results have been found in pre-pandemic studies examining sex and age effects on stress-related exhaustion over time [29]. Both younger age and sex are known risk factors for diminished psychological health (internalizing distress for females, and externalizing distress for males), and it is likely that our results reflect this general effect. The finding that nurses started out with higher levels of burnout than other healthcare professionals is also in line with the wider literature showing that nurses are at high risk for burnout and stress-related illness across the pandemic [4, 30]. There are several explanations for this. For one, it is possible that the work tasks and work environment of nurses have put them at higher risk for the prolonged stress which is believed to precede the development of burnout. One of the most well-known models for explaining the development of burnout is the ‘Job-Demand-Control’ model which posits that high demands in conjunction with low control and ability for decision making (e.g., to organize and manage one’s own workload) poses a risk for strain and stress-related illness [31]. Another model for explaining burnout development is the ‘Effort-Reward’ model which defines dangerous jobs as those exhibiting a mismatch between exerted effort (e.g., due to a high workload) and perceived low levels long-term rewards (e.g. salary, esteem and recognition, job security, promotion possibilities) [32]. Both models can explain the higher levels of burnout among nurses compared to other healthcare professions, given that nurses have high responsibility and demands combined with lower pay and lower control/influence over working conditions. Data from the Norwegian national occupational health surveillance shows that 49% of nurses report low control and autonomy over their work conditions, and 24% report a mismatch in effort-reward; figures that are considerably higher than for other healthcare professions (e.g. physicians, psychologists, dentists) which stand at 27% and 9.5%, respectively [33]. In a meta-analysis looking at burnout among physicians, results suggested that organization-directed interventions were most effective at reducing symptoms of burnout [34]. The dataset at hand prevents us from exploring these mechanisms further, and future studies should design data collections with the ability to further illuminate on organizational and other mechanisms behind nurses’ increased risk of stress-related disorders.

We found that pandemic-related experiences, specifically working with patients with Covid-19 and becoming infected with Covid-19, were associated with increased burnout over time. As for being exposed to patients with Covid-19, this finding might reflect higher overall demands and thus less capacity for recovery. The same could be true for becoming infected with Covid-19. However, we also note that long-term fatigue may arise after Covid-19 infection, as well as other infections, also possibly impacting natural recovery from burnout and other stress-related symptoms [35].

There are several limitations with this study which should be noted. First, there was a considerable decline in the number of participants across the three study waves. This was, however, expected given the nature of the study as an open cohort design. Furthermore, we don’t believe the rate of non-completion at subsequent assessments is a serious threat to the internal validity of the study given that (1) the completers and non-completers did not differ significantly on the major study variable of interest (burnout), and only differed minorly on other study variables, and (2) we managed the missing data using full information maximum likelihood estimation which is widely considered the optimal method for managing missingness. However, as in most longitudinal studies there is a risk of selection bias relating to risk of dropout among participants developing higher symptom levels, for example related to sickness or changes in their work situation. Second, we chose to combine several different professions into the category ‘other frontline HCW’ due to small numbers in these other categories, and it is possible that there are specific professional categories (e.g., prehospital workers, administrative staff) in this group with higher or lower risk than others, respectively. Third, to avoid overburdening the participants and since two items could potentially reflect infection rather than burnout (e.g. feeling sick and tired), two items of the CBI personal burnout subscale were excluded. We acknowledge that this might have impacted the psychometric properties of the measure. Fourth, we caution the reader to keep in mind that the time-dependent predictors (e.g., treatment and death of patients with Covid-19, own or friends, family or collages infection with Covid-19) in the model were coded during the whole study period. While they were only used to predict slope and not starting point, it is possible that some of these variables may have been influenced by earlier burnout symptoms. Finally, although we aimed to recruit widely, there were hospitals and wards who opted out of participating in the study, and there may have been differences between hospitals in which wards participated. Unfortunately, we are not able to obtain this information, but we encourage readers to bear in mind the risk of selection bias both at an organizational and individual level.

This study provides novel information on changes in symptoms of burnout across a prolonged public healthcare emergency. The methodological approach has allowed us to examine both within-person change, which is necessary to understand the intra-individual development of burnout, as well as between-person differences, which allows us to account for factors that could be mitigated at an organizational level. Our findings suggest that while burnout symptoms were fairly stable across the pandemic period that we studied, some HCWs were at increased risk of higher levels of burnout. These were nurses, women, and those of younger age and living alone. Furthermore, certain experiences such as working directly with patients with the disease and being infected oneself can worsen the symptoms of burnout over time. Healthcare managers may want to take extra precautions when staff are exposed to such experiences. These findings may be helpful in planning and responding to future public health emergencies to ensure staff well-being.

## Data Availability

Deidentified individual participant data that underlie the results reported in this article will be available upon reasonable request. In addition, syntax and analytic code can be made available. Data will be available for researchers with a methodologically sound proposal whose proposed use of the data has been approved by an independent review committee. Proposals should be directed to synne.stensland@nkvts.no. Data requestors will need to sign a data access agreement.
